# Chronic Myeloid Leukaemia with isolated massive thrombocytosis and BCR‐ABL1 detection failure using RT‐MLPA (positive RT‐qPCR)

**DOI:** 10.1002/jha2.207

**Published:** 2021-06-04

**Authors:** Lucie Coster, Véronique Mansat‐De MAS, Laetitia Largeaud, Sophie Voisin, Jill Corre, Martin Gauthier

**Affiliations:** ^1^ Service d'Hématologie biologique Centre Hospitalier Universitaire de Toulouse IUCT‐O Toulouse France; ^2^ Université Paul Sabatier Toulouse III Toulouse France; ^3^ Service d'Hématologie clinique Centre Hospitalier Universitaire de Toulouse IUCT‐O Toulouse France

**Keywords:** CML, CML BCR‐ABL, haemostasis, leukaemia cytogenetics, molecular analysis, PCR

A 48‐year‐old woman with no medical history was referred for incidental discovery of thrombocytosis. The full blood count showed discrete anaemia and severe thrombocytosis (haemoglobin concentration 116 g/L, platelets 805 × 10^9^/L), with no increase of any leukocyte (neutrophils 5.1 × 10^9^/L, basophils 0.1 × 10^9^/L) or immature granulocytes. The initial etiological investigation revealed no cause of reactive thrombocytosis, nor *JAK2*, *CALR* or *MPL* mutation (blood samples). Moreover, RT‐MLPA (reverse transcription multiplex ligation‐dependant probe amplification) was considered negative for BCR‐ABL1 in blood sample, because in background noise. While awaiting the results of those investigations, she rapidly developed massive thrombocytosis, with no response to hydroxycarbamide and spontaneous oral cavity bleeding despite no previous bleeding. Low von Willebrand activity (0.08 IU/ml) confirmed an acquired von Willebrand syndrome (AVWS), with decreased factor VIII (0.65), and discordance between von Willebrand activity and von Willebrand antigen (0.60), excluding a trephine biopsy. Concomitantly, platelet count reached 3.699 × 10^9^/L and basophil count reached 0.7 × 10^9^/L (6% of leukocytes), while other haematologic parameters remained within normal range.

Bone marrow smear examination (May–Grünwald–Giemsa) showed hypolobated megakaryocytes (10x objective, red arrow), and 3% of basophils (100x objective, red arrow). Bone marrow karyotype found a t(9;22;17)(q34;q11;p13) translocation, a variant translocation of the classic t(9;22) with a partner chromosome 17, not considered as an additional chromosomal abnormality (ACA), within only 9 over 20 mitoses (with neither i(17)(q10) nor *TP53* deletion) and a low FISH positivity (24% nuclei with *BCR‐ABL1* fusion) (yellow arrows). A diagnosis of chronic myeloid leukaemia (CML) was established and the patient was treated with nilotinib, allowing rapid platelet count normalization (1 month) and subsequent AWVS disappearance (von Willebrand activity, 0.72 and antigen, 0.87). The patient presented a rapid decrease in BCR‐ABL1 transcript and reached both complete cytogenetic and deep molecular response within 3 months (transcript 0.0005%, International scale [IS]), with an undetectable transcript thereafter.

CML presenting with isolated extreme thrombocytosis is a rare but well‐known entity affecting younger patients and more often women. RT‐MLPA failed to detect BCR‐ABL1 and our patient had the lowest BCR‐ABL1 transcript at CML diagnosis in our laboratory (3.6% [IS] RT‐qPCR, comparison with 123 patients) (red square); the BCR‐ABL1 transcript observed was typical e13a2 responsible for the presence of a p210 BCR‐ABL1 protein. This point could raise issues about BCR‐ABL1 transcript value at diagnosis of CML patients presenting with isolated thrombocytosis. Anyway, our case points that, RT‐MLPA, performed on blood sample in isolated thrombocytosis CML suspicion may be questionable and should systematically be checked using RT‐qPCR, which has a better sensitivity. Additionally, we illustrate that, in the case of isolated thrombocytosis, AVWS is frequent, and the presence of hypolobated megakaryocytes and basophils may lead to CML diagnosis and not to essential thrombocythemia as megakaryocytes are big and hyperlobed with no basophils in this latter case.

## DISCLOSURE OF CONFLICTS OF INTEREST

The authors declare they have no conflict of interest.

## CONFLICT OF INTEREST

The authors declare no conflict of interest.

## ETHICS APPROVAL

This article does not contain any studies with human participants performed by any of the authors.

## PATIENT CONSENT

Informed consent was not required in this study.

### AUTHOR CONTRIBUTIONS

Lucie Coster performed cytogenetic analyses; Véronique Mansat‐De Mas and Laetitia Largeaud performed RT‐MLPA and RT‐qPCR analyses; Sophie Voisin performed haemostasis analyses; Véronique Mansat‐De Mas and Jill Corre performed bone marrow smear examination; Martin Gauthier managed the patient; Martin Gauthier, Lucie Coster and Véronique Mansat‐De Mas wrote the manuscript. All authors revised and approved the manuscript.

## Data Availability

The data that support the findings of this study are available from the corresponding author upon reasonable request.

